# Supplementary material for: Interaction of quercetin and epigallocatechin gallate (EGCG) aggregates with pancreatic lipase under simplified intestinal conditions
Source: PLoS One. 2020 Apr 16;15(4):e0224853. doi: 10.1371/journal.pone.0224853 (PMC7161950; doi:10.1371/journal.pone.0224853)
Supplement: S1 Fig — (DOCX) [file pone.0224853.s001.docx]

**Supplementary information**

1. **Molecular weight determination of porcine pancreatic lipase**

**
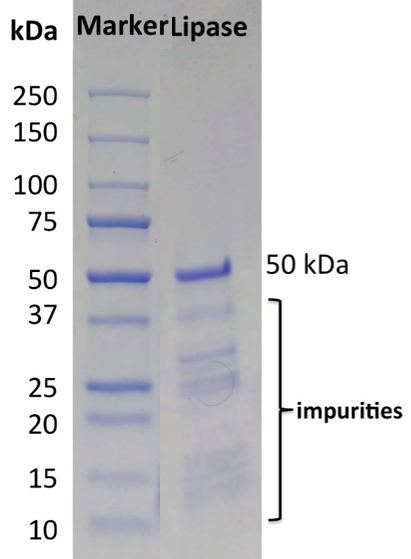
**

**Figure S1.** SDS-PAGE for porcine pancreatic lipase.
